# Microrheology reveals simultaneous cell-mediated matrix stiffening and fluidization that underlie breast cancer invasion

**DOI:** 10.1126/sciadv.abe1969

**Published:** 2021-02-17

**Authors:** Brad A. Krajina, Bauer L. LeSavage, Julien G. Roth, Audrey W. Zhu, Pamela C. Cai, Andrew J. Spakowitz, Sarah C. Heilshorn

**Affiliations:** 1 Department of Chemical Engineering, Stanford University, Stanford, CA 94305, USA.; 2 Department of Bioengineering, Stanford University, Stanford, CA 94305, USA.; 3 Institute for Stem Cell Biology and Regenerative Medicine, Stanford University, Stanford, CA 94305, USA.; 4 Department of Materials Science and Engineering, Stanford University, Stanford, CA 94305, USA.; 5 Department of Applied Physics, Stanford University, Stanford, CA 94305, USA.

## Abstract

Living tissues embody a unique class of hybrid materials in which active and thermal forces are inextricably linked. Mechanical characterization of tissues demands descriptors that respect this hybrid nature. In this work, we develop a microrheology-based force spectrum analysis (FSA) technique to dissect the active and passive fluctuations of the extracellular matrix (ECM) in three-dimensional (3D) cell culture models. In two different stromal models and a 3D breast cancer spheroid model, our FSA reveals emergent hybrid dynamics that involve both high-frequency stress stiffening and low-frequency fluidization of the ECM. We show that this is a general consequence of nonlinear coupling between active forces and the frequency-dependent viscoelasticity of stress-stiffening networks. In 3D breast cancer spheroids, this dual active stiffening and fluidization is tightly connected with invasion. Our results suggest a mechanism whereby breast cancer cells reconcile the seemingly contradictory requirements for both tension and malleability in the ECM during invasion.

## Teaser

Invading breast cancer cells transform the mechanics of their surroundings across a panorama of time scales.

## INTRODUCTION

In living tissues, the mechanical coupling between active cells and their passive extracellular matrix (ECM) forges a unique class of hybrid materials. Active and passive mechanical elements are inseparably linked into a dynamic whole that is greater than the sum of its parts. This reciprocity between active forces and the ECM shapes myriad phenomena relevant to human development, aging, and disease (*1*–*3*). ECM mechanics are now recognized as an essential facet of in vitro tissue models, but commonly relied upon passive mechanic descriptors of ECMs, such as “stiffness,” are inadequate for capturing the marriage of active and passive components that underlies the mechanics of tissues as a whole.

To achieve a quantitative description of tissue mechanics that embodies this duality of active and passive forces, one must embrace the vast hierarchy of time scales involved. Cell-ECM interactions are weaved by processes spanning a formidable expanse of time scales, such as the bend fluctuations of the actin cytoskeleton (10^−6^ to 10^−3^ s) (*4*, *5*), the power stroke of myosin motors (10^−3^ s) (*6*), and the turnover of cell-matrix adhesions (10^0^ to 10^2^ s) (*7*). The molecular relaxations of ECMs span a comparably vast breadth (*8*–*10*). However, in the treatment of ECM mechanics for three-dimensional (3D) tissue models, this breadth of time scales is rarely addressed.

In this work, we develop a methodology to quantify the hybrid active/passive mechanics of in vitro 3D cell culture systems across this full panorama of time scales. We leverage dynamic light scattering microrheology (DLSμR), which interrogates the broadband viscoelasticity of soft materials (*8*, *11*). Applied here to the 3D cell culture systems, DLSμR nondestructively illuminates the hybrid dynamics of the ECM as cell-mediated matrix remodeling unfolds. We develop a force spectrum analysis (FSA) technique to disentangle these dynamics into their underlying time scale–dependent active and thermal contributions.

We harness this FSA to reveal the dynamics of in vitro models of human breast cancer, which is a prototypical disease regulated by tissue mechanics (*12*–*14*). We investigate two models that represent complementary facets of breast cancer progression: tissue remodeling by contractile stromal cells and invasion by breast cancer spheroids. In both systems, our DLSμR and FSA reveal cell-matrix interactions that give rise to rich mechanics involving simultaneous long time scale fluidization and short time scale stiffening. We demonstrate that this coexistence of matrix stiffening and fluidization is a generalizable consequence of nonlinear, time scale–dependent coupling between active forces and the passive viscoelasticity of stress-stiffening networks.

In our spheroid model of breast cancer, these nonlinear mechanics support a reciprocal cell-ECM feedback loop that underlies collective invasion. We find that the ECM composition regulates growth factor–induced invasion by premalignant breast cancer cells. This collective invasion, in turn, requires matrix proteolysis and elevated active cellular forces, which cooperatively drive both time scale–dependent matrix stiffening and fluidization. This dual fluidization and stiffening may serve a mechanism whereby the ECM can satisfy seemingly incongruent functions by supporting transient tension while remaining compliant to the slow process of migration. The multi–time scale nature of these dynamics serves as quintessential illustration of the need for a broadband view of the hybrid mechanics of tissues, and we demonstrate a new technique to quantify those dynamics in living tissues.

## RESULTS

### DLSμR captures cell-mediated changes to ECM dynamics

Our method for interrogating the hybrid active/passive dynamics of tissue-mimetic systems builds on our previously developed DLSμR technique. DLSμR extracts the average dynamics of tracer particles embedded in a material across a broad range of time scales (on the order of 10^−6^ to 10^2^ s) (*8*). We previously demonstrated that when applied to biopolymer matrices, DLSμR reveals time scale–dependent viscoelasticity that is opaque to conventional rheology.

**Fig. 1:**
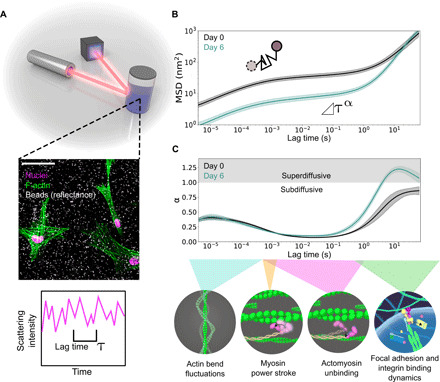
DLSμR overview. (**A**) Schematic of DLSμR. Mammalian cells are encapsulated within a 3D extracellular matrix containing polystyrene tracer particles. Tracer particle dynamics are extracted by measuring light scattering fluctuations across different time scales, τ. The middle inset is a confocal microscopy image of HMFs 6 days after encapsulation in a mixture of col/rBM. F-actin fibers (green false color) were stained with tetramethyl rhodamine B isothiocyanate (TRITC)–phalloidin. Nuclei (magenta false color) were counterstained with DAPI (4′,6-diamidino-2-phenylindole). Polystyrene beads (false-colored white) were visualized by confocal reflectance from a 635-nm laser. Scale bar, 50 μm. (**B**) Tracer particle MSD as a function of lag time obtained from DLSμR measurements with HMFs in col/rBM. Days 0 and 6 indicate the time elapsed since encapsulation. (**C**) Local power-law scaling exponent of particle motion, α, as a function of lag time. The bottom schematic illustrates processes corresponding to different time scale regimes: F-actin bend fluctuations (*4*, *5*), the myosin power stroke (*6*), myosin unbinding lifetimes (*47*, *48*), focal adhesion turnover (*7*), and integrin binding lifetimes (*49*). In (B) and (C), curves represent the geometric and arithmetic means among biological replicates (*n* = 7), respectively. Shaded bands represent 68% confidence intervals of the respective means.

We first harnessed this DLSμR in an in vitro model of ECM remodeling by stromal cells in the breast cancer microenvironment. As a model of the ECM in the vicinity of a breast tumor, we used a mixture of collagen I and reconstituted basement membrane (col/rBM) (*13*, *15*, *16*). The size of our beads (2 μm in diameter) is much larger than the reported pore size of rBM (≈60 nm) (*14*) and the diameter of collagen fibers (≈150 nm) (*17*) at similar concentrations as those used in our experiments. Therefore, our technique probes the continuum viscoelasticity of the matrix. In the absence of cells, DLSμR revealed rich time scale–dependent particle dynamics in this col/rBM matrix (fig. S1), which are qualitatively similar to the hierarchical molecular relaxations of other entangled biopolymer networks (*8*). To investigate the hybrid dynamics arising from active stromal cells, we encapsulated human mammary fibroblasts (HMFs) within the col/rBM and interrogated the mean squared displacement (MSD) of tracer particles embedded within the ECM over 6 days of tissue culture (Fig. 1A).

DLSμR revealed that HMFs reshaped the time scale–dependent ECM dynamics. After 6 days, the absolute magnitude of the MSD and its local power-law scaling behavior MSD ∼ τ^α^ revealed the coexistence of suppressed motion on short time scales and more directed motion on long time scales (Fig. 1, B and C). Over time scales ranging from 10^−5^ to about 10^−1^ s, the magnitude of the MSD was reduced after 6 days of culture by a factor of ≈5. However, the local power-law scaling exponent α remained nearly identical. In contrast, at longer time scales, tracer particle fluctuations after 6 days became more processive, indicated by a larger α that was about twice that of day 0 and superdiffusive (α > 1) on sufficiently long time scales. Superdiffusive motion is not expected from purely thermal fluctuations, where 0 < α < 1, with the lower and upper limits corresponding to purely elastic and purely viscous materials, respectively (*18*). Thus, the data suggest that the particles were driven by active cellular forces. These changes in the dynamics absolutely required the presence of cells (fig. S1) and were abolished by continuous inhibition of F-actin polymerization (fig. S2).

### Tracer particle dynamics contain active and thermal fluctuations

**Fig. 2:**
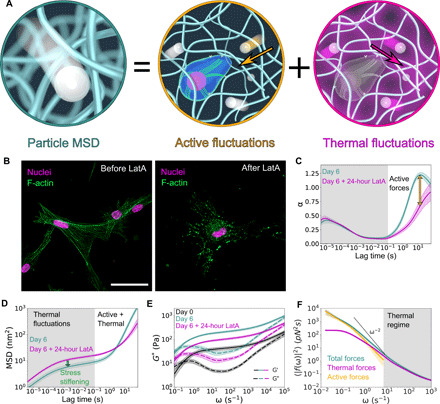
DLSμR captures actin-dependent matrix dynamics and cell-mediated remodeling of viscoelasticity. (**A**) Schematic of the contributions to particle motion from thermal and active fluctuations. (**B**) Confocal microscopy of HMFs cultured in col/rBM for 6 days before and after depolymerization of the F-actin cytoskeleton with latrunculin A (LatA). F-actin fibers (green false color) were stained with TRITC-phalloidin. Nuclei (magenta false color) were counterstained with DAPI. Scale bar, 50 μm. (**C**) Power-law scaling of the MSD, α, before and after latrunculin A treatment. Shading designates regimes where α is independent (gray) or dependent (white) on F-actin polymerization. (**D**) Particle MSD. Shaded regions denote regimes governed by either thermal fluctuations (gray) or a superposition of thermal and active fluctuations (white). (**E**) Shear modulus, G*, of the ECM as a function of angular frequency ω at indicated culture time points. Solid and dashed lines denote the storage modulus *G*^′^ and loss modulus *G*^″^, respectively. (**F**) Force fluctuations at different frequencies ω on day 6. The shaded region designates where thermal forces dominate over active forces. The dashed black line shows the high-frequency scaling of step-like motor forces, 〈∣*f*(ω)∣^2^〉 ∼ ω^−2^. Curves represent the arithmetic (C, E, and F) mean or geometric (D) mean among biological replicates (*n* = 7). Error bands represent 68% confidence intervals of the mean.

These dual time scale dynamics emerge from the hybridization of thermal fluctuations and active cellular forces (Fig. 2A). To tease apart the contributions to particle motion from thermal and active forces, we allowed the HMFs to remodel the col/rBM ECM for 6 days and subsequently depolymerized their actin cytoskeleton by treating with latrunculin A for 24 hours (Fig. 2B). LIVE/DEAD viability measurements demonstrated that this latrunculin A treatment was well tolerated by all cell types studied in this manuscript (figs. S4 to S6). On time scales longer than 10^−1^ s, α was markedly reduced by F-actin disassembly, and the previously observed superdiffusive behavior was abolished (Fig. 2C). However, on shorter time scales, the magnitude of the MSD was increased by a time scale–independent multiplicative factor (Fig. 2D), but the power-law scaling behavior was left untouched. These effects were not observed when the microtubule cytoskeleton was depolymerized with nocodazole (fig. S3), suggesting that F-actin dominated the dynamics we observed.

To reconcile these dichotomous roles of active forces in both suppressing and directing particle motion, we developed an FSA for quantifying the frequency-dependent active and thermal fluctuation spectra of the ECM. Both collagen and rBM are known to exhibit nonlinear stress-stiffening mechanics (*19*–*21*). Our analysis assumes a stress-stiffening matrix that is sustained in a prestressed state by contractile cellular forces. Within this matrix, motion of the embedded particles is driven by both thermal fluctuations and active, cell-generated force fluctuations. In general, we consider a particle with diameter *d* embedded in a viscoelastic fluid with a frequency-dependent differential shear modulus G*(ω), which describes the response of the material to small perturbations superimposed onto the prestress (*22*). Particles are subjected to stochastic forces characterized by a force correlation function 〈*f*(τ)*f*(0)〉 [and corresponding Fourier transform 〈∣*f*(ω)∣^2^ ∣〉]. For small superimposed force fluctuations, such a particle will undergo motion whose Fourier-transformed MSD 〈Δ*r*^2^(ω)〉 is given in (*23*)

$$\langle \Delta r^{2}(\omega )\rangle =\frac{\langle {|f(\omega )|}^{2}\rangle }{{|3\pi dG^{*}(\omega )|}^{2}}$$ (1)

The force spectrum driving the particle can be linearly decomposed into its thermal 〈∣*f*(ω)*_T_*∣^2^〉 and active 〈∣*f*(ω)*_A_*∣^2^〉 contributions, i.e., 〈∣*f*(ω)∣^2^〉 = 〈∣*f*(ω)*_T_*∣^2^〉 + 〈∣*f*(ω)*_A_*∣^2^〉 (Fig. 2A). Thus, if the active force fluctuations generated by the cells exhibit temporal correlations that differ from the underlying thermal force fluctuations of the network, then the power-law scaling behavior of the MSD will be altered on any time scale where active force fluctuations play an appreciable role in driving particle motion. Applying this reasoning, we identified the existence of distinct time scale regimes over which thermal and active fluctuations dominated the motion of the particles (Fig. 2, C and D).

### Fibroblasts actively stiffen the ECM

Our FSA requires an estimate of the frequency-dependent shear modulus G*(ω). To estimate G* on day 6, we assume that F-actin depolymerization completely abolishes active forces, producing motion governed by the generalized Stokes-Einstein relation (GSER) (*18*). We assume that before drug treatment, forces generated by the actin cytoskeleton alter G* by a frequency-independent multiplicative factor due to the ECM’s stress-stiffening response. In the thermal regime, this assumption is supported by our observation that the actin cytoskeleton suppresses the MSD of the particle by a time scale–independent multiplicative factor. At lower frequencies (10^−2^ to 10^2^ s^−1^), this assumption is supported by our macrorheology measurements of the frequency-dependent differential modulus in the presence of an imposed prestress (fig. S7), which revealed that increasing prestress increased G* by a frequency-independent, prestress-dependent multiplicative factor.

This analysis revealed substantial stiffening of the ECM through a combination of transient (reversible) and viscoplastic (irreversible) mechanisms. Over the first 6 days in culture, the fibroblasts globally stiffened the matrix by a frequency-independent factor of about 5 (Fig. 2E), thereby suppressing the underlying thermal ECM fluctuations, as can be observed from the decreased MSD in the short time scale thermal regime on day 6 (fig. S1A). The matrix softened by about half upon F-actin depolymerization, indicating that active stress stiffening reversibly sustained a substantial fraction of this stiffening. We corroborated this stiffening using low-frequency macrorheology measurements on HMF-populated col/rBM (fig. S8).

### Slowly fluctuating active forces mediate high-frequency stress stiffening and low-frequency fluidization

Active forces not only alter ECM viscoelasticity but also compete with thermal fluctuations in driving particle motion. In our measurements, active fluctuations played a dominant role at long time scales and were eclipsed by thermal forces on short time scales (Fig. 2F). In the highest frequency region of the active force regime, the active forces scaled with frequency according to 〈∣*f*(ω)*_A_*∣^2^〉 ∼ ω^−2^. This ω^−2^ scaling is the anticipated high-frequency scaling for a matrix in which fluctuating internal stresses are generated by contractile motor forces that instantaneously release tension after a Poisson distribution of lifetimes (*24*) and has been reported from microrheology of the cytoplasm of living cells (*23*, *25*). At lower frequencies ω < 10^−1^ s^−1^, 〈∣*f*(ω)*_A_*∣^2^〉 adopted a weaker frequency dependence, suggesting that the active forces fluctuated with a broad distribution of correlation times ranging from at least 10 to 10^2^ s.

**Fig. 3:**
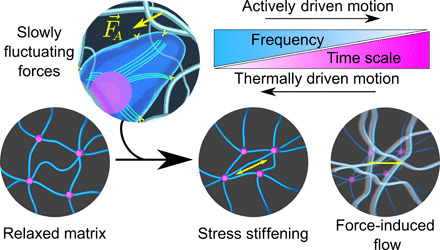
Schematic of time scale–dependent matrix remodeling by fibroblasts. Slowly fluctuating forces generated by contractile fibroblasts alter matrix dynamics in different fashions over different frequency regimes. At high frequencies, thermal fluctuations dominate motion and fluctuating active forces stiffen the matrix through active tension. At low frequencies, fluctuating active forces drive flow of the viscoelastic matrix.

This temporal incongruence between the thermal and active force fluctuation spectra reconciles the dual roles of active forces in both stiffening and fluidization. We reproduced these same qualitative observations with human mesenchymal stromal cells (hMSCs) in col/rBM (fig. S9), which reinforces the general principles underlying this phenomenon. Whenever slowly fluctuating processes control active forces, a high-frequency scaling for active force fluctuations 〈∣*f*(ω)*_A_*∣^2^〉 ∼ ω^−2^ will prevail at short time scales but will be dominated by the broad frequency dependence of the thermal fluctuations of biopolymer matrices on sufficiently short time scales. Together, these competing effects will generally lead to hybrid mechanics in which active forces drive fluidization of the matrix at long time scales where their fluctuations overshadow thermal forces and stiffen the matrix on short times by bearing steady tension on the ECM (Fig. 3).

Our technique involves spatial averaging of particle fluctuations over a scattering volume that is much larger than the size of a cell. However, spatially resolved optical tweezers microrheology measurements have demonstrated that matrix stiffening induced by cellular contraction is highly dependent on distance from the cell (*9*, *21*, *26*). Short-term remodeling of collagen by sparsely seeded breast cancer cells was shown to produce an average stiffening that decayed with distance from the cell such as ∼1/*r*^2^ (*21*). Other studies of contractile cells in collagen also have demonstrated that the greatest stiffening occurs within a distance less than 50 μm (*9*, *26*). However, the spatial distribution of stiffness has been shown to be spatially heterogeneous even at a single distance. In optical tweezers microrheology studies of fibroblasts in collagen, stiffening was concentrated in the pericellular region, but punctate stiffening extended to distances up to about 100 μm (*26*).

In our experiments, cells are seeded at a density of 0.5 cells/nl, corresponding to a characteristic separation length between cells of about 200 μm. Thus, most of the particles measured in our experiments reside at a distance from the cell beyond the previously identified pericellular region. Therefore, the stiffening immediately in the vicinity of the cell may be larger than measured by our spatial averaging technique. Our method also does not capture discontinuous fluctuations in stiffness, which may be relevant to mechanosensing. It is worth noting that the cell density that we use here is about 2 to 10 times larger than the aforementioned optical tweezers studies, and our cultures are permitted to remodel the matrix for much longer than other studies, where remodeling was permitted for less than 28 hours. These differences may result in more extensive long-range matrix stiffening. The large magnitude of effect that we observe here suggests that the stiffening induced by the fibroblasts extends well into the bulk of the material.

Because we do not measure the differential shear modulus of the prestressed ECM directly, our calculation of G* requires an indirect calculation of the thermal fluctuations. This calculation relies on the assumptions described in detail in Materials and Methods. Our calculation assumes that although the system is out of equilibrium, the thermal contribution to the fluctuation spectrum obeys the fluctuation dissipation theorem (FDT), with the differential shear modulus taken as the locally linear response function.

This assumption is not guaranteed but is consistent with optical tweezers microrheology experiments on active biological materials and internally consistent with our results. Studies comparing active and passive microrheology of motor-activated cytoskeleton networks and living cells have demonstrated that the color of the force fluctuation spectrum at thermally dominated high frequencies is consistent with the FDT, despite the fact that the gel exists in a nonequilibrium, stress-stiffened state (*23*, *27*). Similarly, in our system, the high-frequency color of the fluctuation spectrum (but not the magnitude) is identical before and after latrunculin A treatment. This behavior has been previously interpreted using a model in which ever present thermal fluctuations follow the FDT, but the total fluctuation spectrum deviates from the FDT at low frequencies because of the steeper frequency dependence of processive motor forces (*24*).

### Matrix composition regulates morphogenesis and matrix remodeling in a breast cancer spheroid model

We next wielded this FSA to explore the role of hybrid mechanics in regulating the noninvasive to invasive transition of tumor cells. The transformation from premalignant to invasive breast tumors is associated with loss of basement membrane proteins that normally enclose the mammary epithelium and contact of the tumor with the collagen I–rich stromal tissue (*28*). These changes in local ECM architecture at the tumor leading edge are often accompanied by epithelial to mesenchymal transition (EMT), which is thought to promote a more motile, invasive phenotype (*29*). Transforming growth factor–β (TGFβ) is a potent regulator of this EMT process but paradoxically can function as a tumor suppressor or promoter during early or late stages of breast cancer progression, respectively (*30*). Thus, we endeavored to explore how reciprocal feedback between breast cancer cells and the ECM could play a role in bridging this duality in TGFβ signaling.

We chose the H-ras–transformed MCF10AT premalignant human breast cancer model, which is known to form premalignant tumors in mice and to form noninvasive spheroids in 3D Matrigel culture (*13*, *31*). We chose matrices representing either an intact rBM (Matrigel) or a compromised basement membrane consisting of a blend of rBM and porcine collagen I (col/rBM). We note that although our rBM mimics some aspects of the biochemical composition of the native basement membrane, it lacks the structural organization of the basal lamina in vivo. In mammary tissue, the basement membrane is an extremely thin material with a layered organization and planar mechanics that may differ substantially from bulk rBM (*32*, *33*). The process of breaching this uniquely organized material is not represented in our model.

We encapsulated MCF10AT cells as single-cell suspensions in our models of the intact and compromised basement membrane. After performing DLSμR immediately after encapsulation (“day 0”), we treated the cells with or without TGFβ. We allowed 3D morphogenesis to proceed for 6 days before performing an additional DLSμR measurement and fixing cultures for immunofluorescence confocal microscopy.

**Fig. 4:**
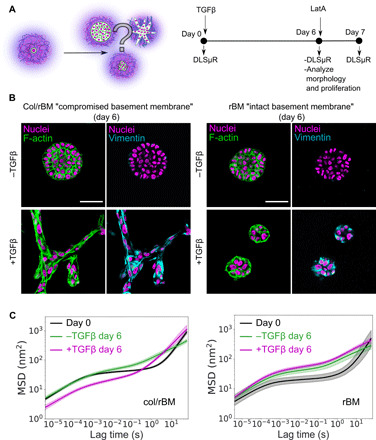
Matrix composition and growth factor signaling jointly regulate 3D invasion and matrix remodeling by MCF10AT premalignant breast cancer cells. (**A**) Schematic of the experiment. (**B**) Confocal microscopy of spheroid morphology after 6 days of culture in either col/rBM (left) or rBM (right) in the presence or absence of TGFβ. F-actin fibers (green false color) were stained with TRITC-phalloidin. Vimentin (cyan false color) was stained by immunofluorescence as an EMT marker. Nuclei (magenta false color) were visualized using an endogenously expressed H2B-GFP fusion protein. Scale bars, 50 μm. (**C**) DLSμR measurements of the impact of TGFβ treatment on particle MSD over the course of tissue culture for cells embedded in either col/rBM (left) or rBM (right). Curves are the geometric mean among independent biological replicates (*n* = 3 to 5). Error bands represent 68% confidence intervals of the geometric mean.

After 6 days of culture, matrix composition commandingly regulated the outcome of TGFβ-mediated EMT. In both matrices, TGFβ treatment promoted classical molecular signatures of EMT, including substantial up-regulation of vimentin (as visualized by immunofluorescence), cadherin expression switching from E-cadherin to N-cadherin [quantified by quantitative reverse transcription polymerase chain reaction (qRT-PCR); fig. S10 and table S1], up-regulation of the EMT “master” transcription factors SNAIL and Zeb1 (fig. S10 and table S1), and up-regulated transcription of matrix metalloproteinases (MMPs) (fig. S11). However, despite similar molecular changes in these EMT markers, TGFβ drove invasion only in col/rBM. In col/rBM, treatment with TGFβ promoted collective invasion of aggregates into the matrix, resulting in highly connected 3D networks formed by interacting spheroids (Fig. 4B). In rBM, treatment with TGFβ resulted in mostly noninvasive clusters (Fig. 4B). These differences in TGFβ-induced invasion starkly contrasted with spheroid morphology in the absence of TGFβ, where both matrices supported proliferation into noninvasive spheroids (Fig. 4B). Our findings are consistent with other in vitro models demonstrating that fibrillar collagen promotes breast spheroid invasion (*16*, *34*).

Similar to contractile stromal cells, the experimental conditions that drove invasion also promoted time scale–dependent, hybrid matrix remodeling, as observed by the particle MSD measured by DLSμR (Fig. 4C). In rBM, cultures in the presence or absence of TGFβ exhibited similar increase in the MSD after 6 days of morphogenesis, suggesting that expression of EMT markers is not sufficient to drive matrix remodeling. In contrast, in col/rBM, treatment with TGFβ induced changes in particle MSD over the full spectrum of time scales interrogated, resulting in suppressed particle dynamics on short time scales and increased particle dynamics on long time scales. Although TGFβ reduced proliferation (fig. S15A) and altered spheroid size (fig. S16), spheroids proliferated to comparable extents in col/rBM and rBM. Thus, the differences in DLSμR we observe between matrices cannot be explained by proliferation.

### Invasive morphogenesis requires active force fluctuations and matrix remodeling

**Fig. 5:**
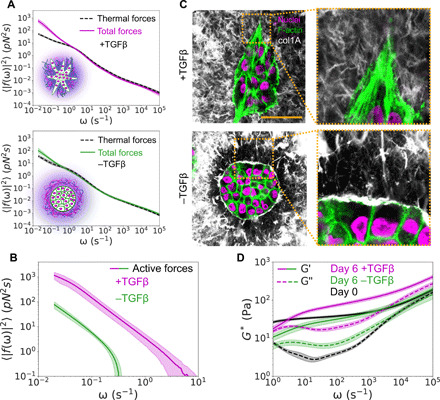
TGFβ promotes active force fluctuations, collagen fiber remodeling, and matrix stiffening by MCF10AT breast cancer cells in a col/rBM ECM. (**A**) Analysis of the thermal contribution to the frequency-dependent force fluctuation spectrum after 6 days in col/rBM. (**B**) Effect of TGFβ on the active force fluctuation spectrum in col/rBM after 6 days. (**C**) Confocal microscopy of collagen fiber organization after 6 days of culture with cells in col/rBM. Collagen I (white false color) was stained by immunofluorescence. F-actin fibers (green false color) were stained with TRITC-phalloidin. Nuclei (magenta false color) were visualized using an endogenously expressed H2B-GFP protein. Scale bar, 50 μm. (**D**) Effect of TGFβ on the frequency-dependent shear modulus G* after 6 days of culture in col/rBM. In (A), (B), and (D), curves represent the arithmetic mean among biological replicates, and bands represent 68% confidence intervals of the means (*n* = 3 for −TGFβ and *n* = 5 for +TGFβ).

These hybrid dynamics driven by invasive spheroids accompanied notable changes in the active force fluctuation spectrum and physical organization of the ECM. FSA revealed that in col/rBM, invasive spheroids exerted active force fluctuations over an order of magnitude greater than noninvasive spheroids (Fig. 5, A and B). Confocal microscopy revealed notable changes in collagen fiber architecture surrounding the spheroids. In the absence of TGFβ, a ring of collagen I aligned parallel to the spheroid surface enclosed the spheroids (Fig. 5C). In the presence of TGFβ, this parallel ring was abolished and fibers exhibited qualitatively more disorganized orientation.

The elevated active force fluctuations and reorganization of the ECM by invasive spheroids coincided with changes in frequency-dependent matrix viscoelasticity. TGFβ not only drove a substantial increase in the magnitude of G* across all frequencies in the thermal frequency regime (Fig. 5D) but also altered the elastic/viscous character of the matrix in a frequency-dependent manner. At higher frequencies, where the modulus took on a predominantly elastic character, the magnitude of *G*^′^ was increased by around a factor of 2 over 6 days. At lower frequencies, the matrix became much more viscous with *G*^″^ approaching *G*^′^. Thus, the fluidization reflected in the particle MSD at long time scales emerged from the cooperative action of both increased active force fluctuations and permanent fluidization of the passive viscoelasticity of the ECM.

### Rho-GTPase–mediated cytoskeletal forces and MMPs are required for matrix remodeling and invasion

We sought to elucidate the molecular mechanisms underlying this correlation between collective invasion, active and thermal fluidization, and matrix stiffening. Thus, we targeted two facets of cell-ECM interactions broadly implicated in both matrix remodeling and 3D migration: Rho-GTPase (guanosine triphosphatase)–mediated cytoskeletal dynamics (*35*) and MMP-mediated ECM degradation (*2*, *36*). To inhibit both protrusive and contractile cytoskeletal forces in TGFβ-treated cells, we treated MCF10AT cells in col/rBM with both a Rac1 inhibitor (EHT 1864) and a ROCK inhibitor (Y27632) throughout the entire culture period. To inhibit matrix proteolysis, we included a broad-spectrum MMP inhibitor (GM6001).

**Fig. 6:**
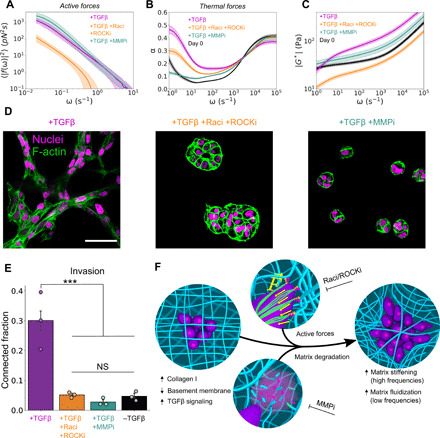
Rho-GTPases and MMPs jointly drive matrix remodeling and invasion. (**A**) Effect of 6 days of either simultaneous Rac1 and ROCK inhibition (+Raci +ROCKi) or MMP inhibition (+MMPi) on the active force fluctuation spectrum of TGFβ-treated MCF10AT spheroids in col/rBM. (**B**) Viscoelastic power-law scaling exponent, α, in different drug treatment groups. (**C**) Magnitude of the shear modulus ∣G*∣ as a function of frequency ω. (**D**) Confocal microscopy of TGFβ and drug-treated spheroids in col/rBM (day 6). F-actin fibers (green false color) were stained with TRITC-phalloidin. Nuclei were visualized using endogenously expressed H2B-GFP. Scale bar, 50 μm. (**E**) Quantification of 3D invasion, as measured by spheroid connectivity (one-tailed *t* test with Benjamini and Hochberg false discovery rate multiple comparisons correction. ***P < 0.001; NS, not significant). (**F**) Summary schematic of cell-mediated remodeling of the ECM. In (A) to (C), curves and bands represent the arithmetic mean and 68% confidence interval of the mean, respectively, among biological replicates (*n* = 5 for no drug and for MMPi cultures and *n* = 8 for Raci/ROCKi cultures). In (E), bars represent the arithmetic mean among independent imaging wells (nine fields of view per well). Error bars represent 68% confidence intervals of the means (*n* = 3). Each data point represents the average of one well.

These pharmacological interventions revealed complementary roles for Rho-GTPases and MMPs in active and thermal fluidization, respectively. Simultaneous Rac and ROCK inhibition, but not MMP inhibition, nearly entirely ablated the increase in active force fluctuations induced by TGFβ (Fig. 6A and fig. S12). In a complementary manner, MMP inhibition abolished fluidization in the thermal spectrum of the ECM. This role of MMPs in thermal fluidization is captured by the thermal contribution to α, which describes the fraction of energy that is stored elastically or dissipated viscously (Fig. 6B). α = 0 corresponds to a purely elastic solid, and α = 1 corresponds to a purely viscous liquid (*18*). After 6 days of remodeling in the presence of TGFβ, α was markedly increased (compared to day 0), particularly on long time scales. Treatment with the broad-spectrum MMP inhibitor abolished this change in α across all frequencies, whereas simultaneous Rac1 and ROCK inhibition only partially attenuated this effect.

Unexpectedly, matrix proteolysis and active forces also cooperatively drove high-frequency matrix stiffening (Fig. 6C). Rac1/ROCK inhibition entirely precluded matrix stiffening, resulting in a matrix that slightly softened over the course of 6 days. After 6 days of culture with the Rac1 and ROCK inhibitors, the matrix viscoelasticity was unaffected by F-actin depolymerization (fig. S13), implying that Rac1 and ROCK jointly dominated transient stress stiffening of the matrix. MMP inhibition also substantially reduced matrix stiffening but resulted in a slightly stiffer matrix than the Rac1/ROCK-inhibited cultures. However, upon F-actin depolymerization at day 6, the ECM in MMP-inhibited cultures softened to a modulus comparable to that of the Rac1/ROCK-inhibited system (fig. S13). This suggests that MMP and Rac1/ROCK inhibition similarly suppressed irreversible viscoplastic remodeling and that residual stiffening in MMP-inhibited cultures was due to transient stress stiffening sustained through Rac1- and ROCK-mediated cytoskeletal tension. The cooperation between cellular contractility and MMPs in matrix stiffening has also been reported by others using active microrheology of the pericellular collagen surrounding fibroblasts (*26*). Thus, these observations may represent a more general feature of contractile remodeling in collagen.

This effect of cell-secreted MMPs notably contrasts with the effect of purified collagenase on matrix viscoelasticity. We performed DLSμR on cell-free col/rBM treated with exogeneous collagenase. As expected, collagenase treatment softened and fluidized the matrix over time, eventually producing a completely fluid matrix (fig. S14). Our findings suggest that matrix degradation facilitates long time scale fibril reorganization, which enables active forces to viscoplastically remodel the network to reconfigure transient network cross-links, thereby stiffening the matrix on time scales shorter than fibril relaxation times.

To investigate potential confounding effects of these drug inhibitors on proliferation, we performed genomic DNA quantification (fig. S15) and microscopy-based cluster size analysis (fig. S16). ROCK and Rac inhibition had no impact on proliferation (fig. S15). However, MMP inhibition substantially reduced proliferation compared to all other conditions and correspondingly produced the smallest average spheroid size (fig. S16). To explore the potential role of reduced proliferation and smaller spheroid size in the MMP-inhibited condition, we preaggregated spheroids in microwell plates (50 cells per spheroid) and encapsulated the spheroids in col/rBM at a cell density comparable to the final time point of the most proliferative condition (col/rBM without TGFβ). Using DLSμR to probe the matrix dynamics, we found that preaggregated spheroids treated with TGFβ and the MMP inhibitor still failed to stiffen and fluidize the matrix after 6 days (fig. S17). These results suggest that the effect of MMP inhibition on matrix remodeling cannot be accounted for by its effect on proliferation and cluster size.

Last, we found that the same molecular mechanisms responsible for matrix fluidization and stiffening were also required for invasion. To quantify collective invasion, we performed confocal microscopy on day 6 and quantified the 3D connected structures formed (Fig. 6, D and E). Unlike the control TGFβ-treated cells, which formed highly connected invasive networks, broad-spectrum MMP inhibition produced small cell aggregates that were isolated from one another. Likewise, Rac1/ROCK inhibition substantially decreased the presence of invasive, connected networks. Either ROCK or Rac inhibition alone was insufficient to ablate active force fluctuations and invasion (fig. S10), which is consistent with reports that migrating cells can use a bistable switch between ROCK- and Rac-mediated migration modes (*35*, *37*). These results suggest that cell-mediated changes to the time scale–dependent hybrid active/passive mechanics of the ECM are tightly interwoven with the process of collective invasion.

## DISCUSSION

Our results demonstrate an unexpected role for MMPs in regulating matrix stiffness. Matrix stiffening is often viewed as the outcome of an imbalance between the opposing actions of matrix building and matrix degradation (*38*–*40*). This concept of opposing processes suggests that MMP fluidization and matrix stiffening cannot occur simultaneously within the same matrix. However, we show that MMPs are required for both stiffening and fluidization and that these outcomes exist simultaneously by occupying different frequencies of the viscoelastic spectrum. This may serve as a potential mechanism to reconcile a paradox of breast cancer invasion: Matrix stiffness drives the invasive phenotype (*12*–*14*), but the matrix can create a barrier to 3D migration (*36*, *41*). Simultaneous matrix stiffening and fluidization may enable migrating cells to generate cytoskeletal tension on time scales shorter than the turnover of force-bearing mechanisms while slowly remodeling the matrix to forge a path for migration. This mechanism relies upon the rich time scale dependence of both cellular forces and the molecular relaxation spectra of naturally derived ECMs and can only be adequately articulated by capturing both active and thermal components of ECM dynamics over a broad cascade of time scales.

It has been proposed that oncogenic mutations that disrupt normal epithelial TGFβ signaling may enable switching between its tumor suppressor and tumor promoter activities (*30*). Our study highlights the possibility that changes in the ECM may also mediate TGFβ role switching during tumor progression. In particular, we demonstrate that in a cell line that is susceptible to the antiproliferative effects of TGFβ, matrix composition can determine the impact of TGFβ on matrix remodeling and invasion. Our results suggest that breaching the basement membrane could potentiate a TGFβ-dependent feed-forward loop that drives further tissue remodeling, fibrosis, and invasion, which may support tumor progression.

More generally, our results establish DLSμR and our FSA as powerful tools for interrogating the hybrid mechanics of 3D tissue models. DLSμR is complementary to existing techniques such as optical tweezers–based microrheology, which has been combined with passive microrheology to extract the thermal and active fluctuation spectrum of active motor gels (*27*) and the cytoplasm of living cells (*9*, *23*) or used to map the spatially varying mechanics of ECMs populated with contractile cells (*9*, *21*). Similar to other probe-based optical techniques, DLSμR requires signal from the embedded particles to dominate over scattering from the underlying material (*42*, *43*), and it remains challenging to extract absolute viscoelasticity from endogenous tissue scattering (*44*, *45*). Thus, broadband microrheology of thick, highly scattering tissues represents a crucial challenge for the future. Although the ensemble averaging inherent to our technique sacrifices the ability to spatially map material properties, the high statistical power of DLSμR and its facile implementation on a commercial benchtop platform offer the opportunity to rapidly characterize ECM dynamics in a manner that is broadly accessible to a range of matrix biology, biomaterials, and biophysics researchers. We envision that insights gained from examining the broadband dynamics of the ECM in vitro will inform the design of new models of human tissues that more faithfully recapitulate native physiology and lead to improved models of human development, aging, and disease.

## MATERIALS AND METHODS

### Dynamic light scattering microrheology

DLSμR measurements on cell-populated ECM hydrogels were performed as previously described for cell-free hydrogels (*8*). Measurements were performed in the single-scattering regime using a commercial benchtop dynamic light scattering instrument (Malvern Zetasizer Nano ZS) equipped with a 633-nm laser in 173° backscattering detection. Scattering intensity autocorrelation functions were collected for 30 min at 37°C. To correct for broken ergodicity using our previously described correction procedure, the scattering intensity was measured at 24 positions in the cuvette for 10 s each. The raw scattering intensity autocorrelation function was transformed to obtain the ensemble-averaged particle MSD using a custom analysis software written in Python.

To confirm that measurements were performed in the single-scattering regime, we performed DLS on bead suspensions in water at the same concentration used for all microrheology measurements [0.25% (w/v)]. We conducted CONTIN and second-order cumulant particle size analysis on these data using the known viscosity of water. The scattering correlation function was consistent with single scattering from a low-polydispersity suspension of particles with mean size close to that reported by the manufacturer.

All DLS measurements were performed using disposable 40-μl cuvettes (Malvern ZEN0040) sterilized with 70% ethanol. Before filling, a 0.8-mm-thick silicone isolator was secured at the bottom of the cuvette to ensure filling to the height of the laser spot throughout cell culture. For all measurements on cell-populated matrices, cells were enzymatically dissociated from tissue culture flasks (with trypsin-EDTA or TrypLE express, as specified below), manually counted with a hemocytometer, pelleted by centrifugation, and resuspended as a single-cell suspension (5 × 10^5^ cells/ml for all experiments) in ice-cold ECM precursor solution containing 0.25% (w/v) 2.0-μm PEGylated polystyrene beads [Polysciences, no. 1837-10; PEGylated as described previously (*8*)]. To prepare col/rBM precursor solution, a solution (3.0 mg/ml) of acid-solubilized porcine tendon collagen I (Nitta Gelatin Cellmatrix, no. 631-00651) was mixed on ice with 10× concentrated Dulbecco’s modified Eagle’s media (DMEM) (Sigma-Aldrich, no. D2429-100ML) and collagen neutralization buffer (26 mM sodium bicarbonate, 0.05 N of NaOH, and 200 mM Hepes) at a ratio of 8:1:1 to form a neutralized solution of collagen I (2.4 mg/ml). This collagen solution was mixed on ice 1:1 with reduced growth factor, phenol red–containing Matrigel (Corning, no. 354230) to form a solution with collagen I (1.2 mg/ml) and basement membrane proteins (about 4 mg/ml). For experiments with pure rBM, Matrigel was used directly as provided by the manufacturer. To ensure suspension as single cells, the cell/ECM precursor solution was slowly pipetted up and down 20 times on ice. Following resuspension, 40 μl of cell-populated ECM solution was transferred to a disposable cuvette (Malvern ZEN0040), and the cuvette was placed in a humidified tissue culture incubator at 37°C and 5% CO_2_ for 30 min to allow the ECM to gel. Three hundred microliters of cell culture media (see the “Cell culture” section for cell type–specific formulation) was added on top of the gel after 30 min. The first DLSμR measurement on day 0 was performed immediately after adding warm media on top of the gel. Between DLSμR measurements, the cuvettes containing the cell-populated ECM hydrogel were maintained in the humidified tissue culture incubator at 37°C and 5% CO_2_.

### Calculation of G*

The materials described in our work exist in a prestressed, out-of-equilibrium state and a nonlinear mechanical regime. For our purposes, we define G* of the prestressed material as the response of the material to small deformations superimposed onto the prestressed state, i.e., the differential shear modulus. In macrorheology, this is analogous to the modulus measured by applying a steady prestress to the material and superimposing a small oscillatory stress, which produces a linear strain perturbation for small superimposed stresses (*22*). In active microrheology of a nonequilibrium material, this is analogous to measuring the response of an internally stressed material to a small external stress (*23*, *24*). Our calculation of G* requires an indirect estimation of the thermal contribution to the force spectrum that relies on the following assumptions:

1) The ECM exists in a prestressed pseudo–steady state that can be described by a differential shear modulus, G*. The differential shear modulus describes the response to small fluctuations superimposed onto the prestress.

2) Thermal and active force fluctuations are linearly superimposed onto the pseudo–steady state prestress. The strain response of the material to each of these fluctuations can be described by the locally linear response of the differential shear modulus.

3) The thermal contribution to the fluctuation spectrum is assumed to be consistent with the FDT and GSER. In the GSER, the equilibrium linear shear modulus is replaced with the differential shear modulus of the prestressed material.

4) At sufficiently high frequencies, thermal fluctuations dominate over active force fluctuations. The pseudo-steady prestress maintains the material in a nonequilibrium state.

5) After treatment with latrunculin A, the material is no longer in a prestressed state, and particle fluctuations are due entirely to thermal fluctuations.

6) The differential shear modulus that is produced as a result of the prestress differs from the linear shear modulus of the unstressed material by a frequency-independent multiplicative factor.

Let 〈Δ*r*^2^(*t*)〉*_A_* denote the particle MSD in the presence of active cytoskeletal tension, and let 〈Δ*r*^2^(*t*)〉_0_ denote the particle MSD after inhibition of cytoskeletal tension. We define the critical time scale for transition to the thermally dominated regime, τ*_c_*, to be the time such that for *t* < τ*_c_*

$${\langle \Delta r^{2}(t)\rangle }_{A}=C{\langle \Delta r^{2}(t)\rangle }_{0}$$ (2)

where *C* is a constant multiplicative factor. This is equivalent to the time scale such that

$$\alpha_{A}(t)=\alpha_{0}(t)$$ (3)

for *t* < τ*_c_*.

We identify the multiplicative factor *C* by first determining τ*_c_* by comparison of α*_A_* and α_0_ and then minimizing the sum of squared residuals with respect to *C* between 〈Δ*r*^2^(*t*)〉*_A_* and *C*〈Δ*r*^2^(*t*)〉_0_ in logarithmic space over the time interval *t* < τ*_c_*.

From assumptions 3 to 5, it follows that for ω > 1/τ*_c_*

$$G^{*}(\omega )=\frac{k_{B}T}{\pi ai\omega {\langle \Delta r^{2}(\omega )\rangle }_{A}}=\frac{1}{C}\frac{k_{B}T}{\pi ai\omega {\langle \Delta r^{2}(\omega )\rangle }_{0}}=\frac{1}{C}G_{0}^{*}(\omega )$$ (4)

where *a* is the particle radius (1 μm).

Last, we apply assumption 4 to extend this relation to all frequencies. Thus, we compute the differential shear modulus of the prestressed materials as

$$G^{*}(\omega )=\frac{1}{C}\frac{k_{B}T}{\pi ai\omega {\langle \Delta r^{2}(\omega )\rangle }_{0}}$$ (5)

for all ω.

### Calculation of 〈∣*f*(ω)*_A_*∣^2^〉

To determine the active force fluctuation spectrum, we use G* obtained as above and apply the generalized linear Stokes relation for a particle in an arbitrary force fluctuation field, which yields the total force fluctuation spectrum

$$\langle {|f(\omega )|}^{2}\rangle ={\langle \Delta r^{2}(\omega )\rangle }_{A}{|6\pi aG^{*}(\omega )|}^{2}$$ (6)

We assume that the active and thermal force fluctuation spectra are linearly superposed, as per assumption 1 in the preceding section

$$\langle {|f(\omega )|}^{2}\rangle =\langle {|f{\left(\omega \right)}_{T}|}^{2}\rangle +\langle {|f{\left(\omega \right)}_{A}|}^{2}\rangle$$ (7)

In accordance with assumption 3 from the preceding section, we obtain the thermal contribution to the force from the FDT and the generalized Stokes friction

$$\langle {|f{\left(\omega \right)}_{T}|}^{2}\rangle =36k_{B}T\pi aG^{\prime \prime }(\omega )/\omega$$ (8)

Last, we obtain the active force fluctuation spectrum, 〈∣*f*(ω)*_A_*∣^2^〉, by combing Eqs. 7 and 8.

### Cell culture

Primary HMFs were purchased from ScienCell Research Laboratories (catalog no. 7630) and used for DLSμR between passage 4 and 6. For 2D expansion, HMFs were cultured on poly-l-lysine–coated tissue culture polystyrene flasks in a humidified incubator at 37°C and 5% CO_2_ and maintained in fibroblast growth medium-2 (FGM-2) (Lonza, no. CC-3132) containing 2% fetal bovine serum (FBS), 0.0005% recombinant human insulin, basic human fibroblast growth factor B, and gentamycin/amphotericin B. Cells were passaged by dissociation with 0.05% trypsin-EDTA (Thermo Fisher Scientific, no. 25300054) upon reaching 70 to 90% confluence, and media was changed every other day. For DLSμR experiments, the FGM-2 media was supplemented with recombinant human TGFβ1 (10 ng/ml) (Thermo Fisher Scientific, no. PHG9204) to promote matrix remodeling, and media was changed daily.

H-Ras–transformed MCF10AT cells expressing an H2B-GFP (green fluorescent protein) fusion protein were a gift from J. Liphardt’s laboratory (Stanford University). The cells were maintained, as previously described (*46*). Briefly, cells were expanded on tissue culture polystyrene and maintained in phenol red–containing DMEM/F12 (Thermo Fisher Scientific, no. 10565018) supplemented with 5% normal horse serum (Thermo Fisher Scientific, no. 16050122), recombinant human EGF (20 ng/ml) (no. PHG0311), hydrocortisone (0.5 mg/ml; Sigma-Aldrich, no. H0135), cholera toxin (100 ng/ml; Sigma-Aldrich, no. C8052), recombinant human insulin (10 μg/ml; Sigma-Aldrich, no. I9278), and penicillin-streptomycin (1%; Thermo Fisher Scientific, no. 15140122). Cells were passaged every other day by dissociation with TrypLE Express (Thermo Fisher Scientific, no. 12604013) upon reaching 70 to 90% confluence. For DLSμR experiments, cells were cultured in the above MCF10A growth media and media was changed daily. For experiments with TGFβ, recombinant human TGFβ1 (10 ng/ml; Thermo Fisher Scientific, no. PHG9204) was added to the cell culture media after the first DLSμR measurement on day 0.

hMSCs derived from bone marrow were purchased from Lonza. hMSCs were expanded on tissue culture polystyrene with high-glucose DMEM with GlutaMAX (Thermo Fisher Scientific, no. 10566016) supplemented with 10% FBS (Thermo Fisher Scientific, no. 26140079) and 1% penicillin-streptomycin (Thermo Fisher Scientific, no. 15140122) in a humidified incubator at 5% CO_2_ and 37°C. DLSμR experiments were performed with hMSCs at passage 7.

### Pharmacological intervention

All pharmacological interventions were performed by diluting drugs from a 1000× concentrated dimethyl sulfoxide (DMSO) stock solution into warm culture media and by performing a fresh media change. F-actin depolymerization was achieved by treating with 500 nM latrunculin A for 24 hours. ROCK, MMP, or Rac1 inhibition was performed by adding Y-27632 (10 μM; Cayman Chemical, no. 10005583), GM6001 (30 μM; Cayman Chemical, no. 14533), or EHT1864 (5 μM; Cayman Chemical, no. 13196), respectively, to the media after the first DLSμR measurement on day 0. Microtubule depolymerization was performed by treating with nocodazole (10 μM; Sigma-Aldrich, no. M1404) for 24 hours.

### Confocal microscopy and immunofluorescence

High-resolution confocal microscopy was performed using a Leica SPE confocal microscope with either a 40× or 63× oil immersion objective. Cell-populated hydrogels used for confocal microscopy were cast in 4-mm-diameter, 0.8-mm-thick silicone molds that were plasma bonded to glass coverslips. Gel-filled molds were placed in 24-well tissue culture plates and overlaid with 700 μl of complete growth media. Confocal microscopy was also performed using hydrogels that were transferred from the DLSμR cuvettes and sandwiched between glass coverslips to confirm that qualitative differences were not observed between the different culture geometries.

Samples were prepared for fluorescence microscopy by fixation with 4% paraformaldehyde and 0.1% glutaraldehyde (glutaraldehyde included to prevent Matrigel depolymerization) in phosphate-buffered saline (PBS) for 30 min at room temperature. The samples were washed once with 200 mM glycine in PBS for 15 min, followed by three 15-min washes with PBS. Cell membranes were permeablized with 0.1% Triton X-100 in PBS (PBST) for 1 hour. Blocking was performed by incubating with 10% normal goat serum in PBST for 3 hours at room temperature. For immunofluorescence, primary antibodies against vimentin (1:200; Cell Signaling Technology, no. 5741), collagen I (1:100; Pierce, no. PA1-85317), or β-tubulin (1:100; Cell Signaling Technology, no. 2128) were diluted with 0.05% Triton X-100, 0.1% Tween-20, and 0.1% bovine serum albumin (BSA) in PBS. Primary antibodies were incubated either overnight at 4°C (for vimentin and microtubules) or for 2 days at 4°C (for collagen I). Samples were washed three times in PBST for 20 min. Secondary antibody staining was performed using goat anti-rabbit Alexa Fluor 647 (1:400; Invitrogen, no. A32733) or goat anti-rabbit Alexa Fluor 488 (1:400; Invitrogen, no. A11034) diluted in 0.05% Triton X-100, 0.1% Tween-20, and 0.1% BSA in PBS, followed by three 20-min washes with PBST. Nuclei and F-actin, respectively, were stained by incubation with DAPI (1 μg/ml; 4′,6-diamidino-2-phenylindole) and phalloidin–tetramethyl rhodamine B isothiocyanate (0.2 μg/ml) (phalloidin-TRITC; Sigma-Aldrich, no. P1951) in PBST for 1 hour at room temperature, followed by three 20-min washes in PBST. For samples in which only nuclei and F-actin were stained, no blocking was performed, and staining was performed immediately after permeablization. Last, the samples were mounted onto a no. 1 coverslip using ProLong Gold Anti-Fade (Thermo Fisher Scientific, no. P36930) and allowed to cure for 24 hours before imaging. Polystyrene beads were visualized by confocal reflectance using a 635-nm laser.

### LIVE/DEAD microscopy assay and analysis

Viability was evaluated by staining with calcein AM to label live cells and ethidium homodimer to label dead cells using a LIVE/DEAD cytotoxicity kit (Invitrogen, no. L3224). All cell types were encapsulated in col/rBM at 5 × 10^5^ cells/ml and cast into 4-mm-diameter circular silicone molds (10 μl per mold, prepared as described in “confocal microscopy and immunofluorescence”). Each treatment condition was performed in quadruplet. Cells were cultured for 6 days (MCF10ATs or HMFs) or 4 days (hMSCs) in complete growth media (as described in cell culture). Media was changed every other day. For MCF10AT cells, TGFβ (10 ng/ml; Thermo Fisher Scientific, no. PHG9204) was included in the complete growth media. Media were then exchanged for complete growth media containing latrunculin A (500 nM) or DMSO vehicle (1000× dilution), and cells were treated for 24 hours. After 24 hours, cells were stained with calcein AM (2 μM; 2000× dilution of DMSO stock) and ethidium homodimer (4 μM; 500× dilution of DMSO stock) in DMEM by incubating for 45 min at 37°C. Wells were then washed two times with PBS (containing calcium and magnesium) for 5 min each. For hMSCs and HMFs, gels were imaged directly in the wells (submerged in PBS) using a 10× air objective. To image MCF10ATs, molds containing the gels were inverted onto no. 1.5 coverglass and imaged using a 20× air objective. Imaging near the top of the gels containing MCF10ATs was necessary because of diffusion limitations of the calcein AM at high cell densities. Z stacks were collected with 10-μm spacing between slices (nine slices in total).

The number of live and dead cells in each stack was quantified with custom scripts written in Python using the scikit-image package. Binary images of the live channel were obtained using Li’s minimum cross entropy thresholding method (applied slice wise). Binary images of the dead channel were obtained using Yen’s method (applied on the full z stack). After three successive dilations of the binary dead image to avoid overcounting blebbing nuclei, all 3D connected objects were computed for both channels. Small objects (50 voxels for the dead channel and 100 voxels for the live channel) were removed, and the number of distinct connected objects in each channel was computed as the number of live or dead cells, respectively.

### Macrorheology of ECM nonlinear stress stiffening

Macrorheology experiments were performed using an AR-G2 stress-controlled rheometer with a diameter of 20 mm, 1° angle cone, and plate geometry at 37°C with an aqueous solvent trap to prevent sample dehydration. Ice-cold ECM precursor solution was transferred to the geometry and allowed to gel at 37°C while oscillating at 1 rad/s and 5% strain to confirm steady-state gelation before measurements. The linear shear modulus was obtained by performing a frequency sweep while oscillating at 5% strain with no prestress. An amplitude sweep was performed to confirm that this represents the linear viscoelastic regime. To determine the nonlinear differential shear modulus, we followed a previously reported method for nonlinear differential shear modulus measurements (*22*). Briefly, a steady prestress was applied to the sample and a small oscillation with a stress amplitude equal to 1/10 of the prestress was superimposed onto the steady prestress. Within a given sample, measurements were collected for each reported prestress in order of increasing prestress.

### Macrorheology of HMF-mediated ECM stiffening

HMFs were cultured in 90 μl of col/rBM hydrogels loaded into 8-mm-diameter, 2.5-mm-thick circular silicone molds. Rheometry was performed using an AR-G2 stress-controlled rheometer with an 8-mm-diameter parallel plate. After 6 days of culture, the gels were gently dislodged from the molds with a spatula and transferred to the rheometer plate. The loading gap was adjusted until conformal contact between the gel and the parallel plate geometry was achieved. PBS solution was added around the geometry to prevent sample dehydration. The linear shear modulus was obtained by performing a frequency sweep while oscillating at 5% strain at 37°C. Measurements were performed on three biological replicates.

### MCF10AT spheroid connectivity and size analysis

MCF10AT spheroid connectivity and size analysis was performed with a custom analysis script written in Python using the scikit-image Python package and the ndimage module from SciPy. All cells were stained for F-actin with phalloidin-TRITC, and nuclei were visualized using the endogenously expressed H2B-GFP protein. Images for analysis were collected using a 40× oil objective on a Leica SPE confocal microscope. Z stacks were collected at 5-μm intervals throughout the sample, and a 3 × 3 grid of contiguous Z stacks was stitched together for each biological replicate. Stitched images in three different areas of the sample were collected for each replicate. Imaging was performed on three biological replicates. Shape analysis was performed by first applying a median filter to both the nuclear and F-actin channels and then thresholding both filtered channels using Otsu’s method. A single binary channel was obtained by taking the union of the nuclear and F-actin thresholds. 3D connected structures were identified from the binary image using the morphology.label() function in scikit-image. Objects with volumes less than 500 voxels were removed. Projected areas were determined by taking projections of each 3D connected object. Connectivity was computed as $C=\frac{\Sigma_{i}(V_{i}^{2})}{{\left(\Sigma_{i}V_{i}\right)}^{2}}$, where *V_i_* is the volume of the *i*^th^ connected structure, and the summation is taken over all connected structures. This metric represents the probability that any two unit volumes belong to the same 3D connected structure.

### mRNA gene expression analysis

mRNA expression for EMT markers by MCF10AT spheroids was performed by qRT-PCR. Biological replicates for qRT-PCR were cultured as 40 μl of hydrogel in 8-mm-diameter, 0.8-mm-thick circular silicone molds. To collect cell lysates for qRT-PCR, gels were transferred to TRIzol reagent (Thermo Fisher Scientific, no. 15596026) and disrupted with a probe sonicator. mRNA samples were purified by phenol-chloroform extraction using phase-lock gels (5PRIME), followed by isopropanol precipitation and resuspension in nuclease-free water. One microgram of RNA was reverse transcribed using a high-capacity cDNA reverse transcription kit (Applied Biosystems, Thermo Fisher Scientific, no. 4368813). The resultant reverse transcription reaction was diluted 50-fold with nuclease-free water, and qPCR was performed by mixing 5 μl of the diluted reverse transcription reaction with 10 μl of Fast SYBR Green Master Mix (Applied Biosystems, Thermo Fisher Scientific, no. 4385612) containing 0.09 μM of each primer. The reaction mixture was run on Applied Biosystems StepOnePlus Real-Time PCR System, and mRNA expression was analyzed using the Δ*C*_T_ method. A total of four biological replicates were used for each experimental condition. Primer pairs were purchased from Integrated DNA Technologies and are listed in table S2.

### MCF10AT proliferation assay

Relative proliferation of MCF10AT cells in 3D ECMs was performed by fluorometric quantification of total genomic DNA using PicoGreen dye. Each biological replicate (*n* = 4) was cultured in a 10 μl of hydrogel cast in a 4-mm-diameter, 0.8-mm-thick circular silicone mold. DNA samples were collected by transferring each gel to 200 μl of lysis buffer [20 mM tris-HCl, 150 mM sodium chloride, and 0.5% Triton X-100 (pH 7.4)] and sonicating with a probe sonicator until the gel was completed disrupted. Five microliters of lysis solution was diluted with 20 μl of TE buffer [10 mM tris-HCl and 1 mM EDTA (pH 8.0)] and loaded into a 384-well black-bottom plate. PicoGreen dye (Quant-iT PicoGreen, Thermo Fisher Scientific, no. P11496) was diluted 200-fold with TE buffer, and 30 μl of diluted reagent was added to each well. The sample plate was mixed by shaking and incubated in the dark for 10 min before collecting spectrophotometry measurements. Fluorescence was read using a well-plate spectrophotometer by excitation with 488-nm light and collecting emission at 520 nm. A standard curve to identify the linear regime was obtained by directly lysing 10 μl of cell suspensions containing defined cell concentrations (determined by manually counting with a hemocytometer). Any samples that were outside of the linear regime identified by the standard curve were diluted twofold, and the absolute fluorescence was corrected accordingly.

### Purified collagenase DLSμR time course assay

Collagenase time course assays were performed using type IV collagenase (Gibco, no. 17104019) dissolved freshly in DMEM/F12 at 10 mg/ml. Col/rBM hydrogels containing 2.0 μm of polystyrene beads were prepared as described in the “Dynamic light scattering microrheology” section above. After pH neutralization, hydrogels (40 μl) were cast in disposable cuvettes and incubated for 30 min at 37°C. After gelation, 300 μl of collagenase-containing media (10 mg/ml) was added on top of the gel. Immediately after addition of the collagenase, DLSμR measurements were performed continuously for 90 min at 37°C, with each time point representing an average of the correlation function over a 10-min interval. After 90 min, a scattering intensity measurement was performed over 24 measurement positions (10 s each) to correct for broken ergodicity in all time points. Time courses were performed on three full reproductions of the experiment.

### Preaggregation of MCF10AT spheroids

To test whether preaggregation of spheroids can rescue matrix remodeling in MMP-inhibited spheroids, aggregates were preformed in low-adhesion 400-μm microwell plates (STEMCELL Technologies AggreWell 400 24-well plates) with 50 cells per spheroid. To prevent adhesion to the plate, microwell plates were incubated with 0.5% BSA in PBS at 37°C for at least 6 hours. The BSA solution was completely aspirated from all microwells. A single-cell suspension of MCF10AT cells in complete MCF10AT media. To each well of the 24-well plate (containing 1200 microwells per well), 1 ml of a suspension containing 6 × 10^4^ cells/ml was added. The plate was centrifuged twice at 200*g* for 5 min each. Plates were then incubated at 37°C for 16 hours to allow aggregates to form. After 16 hours of aggregation, spheroid formation was confirmed by phase contrast microscopy. Spheroids were removed from the microwells by pipetting up and down with 1 ml of media. This was repeated with a fresh 1 ml of media until nearly all spheroids were removed. Spheroids were collected by centrifugation at 200*g* for 3 min and then resuspended in col/rBM containing 2.0 μm of beads at a density of 1.2 × 10^5^ spheroids/ml (i.e., 6 × 10^6^ cells per ml). Forty microliters of each hydrogel solution was cast into disposable cuvettes, and DLSμR was performed on day 0, day 6, and after 24 hours of latrunculin A treatment beginning on day 6, as described for other conditions. The MMP inhibitor GM6001 (30 μM; Cayman Chemical, no. 14533) and TGFβ (10 ng/ml; Thermo Fisher Scientific, no. PHG9204) were included throughout the duration of culture. For confocal microscopy, spheroid-populated hydrogels were cast in 4-mm-diameter circular silicone molds. On day 6, cells were fixed, stained using TRITC-phalloidin to visualize F-actin, and imaged by confocal microscopy using a 40× oil immersion objective, as described in confocal microscopy and immunofluorescence.

### Biological replication and reproducibility

Replicates (*n*) represent distinct cell/gel suspensions cast in either separate cuvettes (for DLSμR) or separate circular molds (for confocal microscopy and qPCR). For most DLSμR experiments, replicates were collected across multiple cell passages collected on separate days (with each passage contributing at least two replicates; totally, a minimum of three replicates, as indicated). In some cases, all replicate gels were prepared from the same cell passage. The number of distinct cell passages from which replicates were pooled for DLSμR experiments are as follows (by experimental condition): HMFs, 3; MCF10AT +TGFβ in col/rBM, 2; MCF10AT −TGFβ in col/rBM, 2; MCF10AT +MMPi, 2; MCF10AT +Rocki +Raci, 2; MCF10AT +ROCKi, 3; MCF10AT +Raci, 1; and MCF10AT −TGFβ in rBM, 1. For confocal microscopy–based quantification of morphology, all conditions and replicates were prepared from the same cell passage. Qualitative observations of invasive morphology were confirmed for all conditions in at least three separate passages.

### Statistical Analysis

Data are represented as either the arithmetic mean or geometric mean (where indicated), with shading or error bars indicating 68% confidence intervals of the mean. Confidence intervals were computed by bootstrapping with replacement using custom scripts written in Python. With one exception (described below), statistical significance was evaluated using a one-sided *t* test with multiple comparisons corrected for using a Benjamini and Hochberg false discovery rate correction using R (*P < 0.05, **P < 0.01, and ***P < 0.001; NS, not significant). In cases where equal variances were not supported by the data, unpooled variances were used, as indicated. Otherwise, pooled variances were used.

To evaluate the difference in fold induction of gene expression upon TGFβ treatment between ECMs, *t* test was not used since this does not represent simply a difference of normally distributed variables (it is a difference of differences). Instead, a two-sided parametric bootstrap *t* hypothesis test was performed using a custom analysis script written in Python. Briefly, for a given gene, the studentized bootstrap distribution of log fold changes in expression (with TGFβ versus without TGFβ) was computed for each ECM by parametrically sampling from the student *t* distribution (10^5^ bootstrap samples). The empirical distribution of differences in log fold changes (induced by TGFβ) between ECMs was obtained by forming pairwise differences between the two bootstrap distributions. Confidence intervals, CI, representing different significance levels were formed (*P* = 1 − CI), and the largest confidence interval not encompassing zero was taken as the significance level. Multiple comparisons were then corrected for using the Benjamini and Hochberg false discovery rate correction in R.

## SUPPLEMENTARY MATERIALS

Supplementary material for this article is available at http://advances.sciencemag.org/cgi/content/full/7/8/eabe1969/DC1

## Appendix

View/request a protocol for this paper from *Bio-protocol*.
